# The Predictors of Target Lesion Revascularization and Rate of In-Stent Restenosis in the Second-Generation Drug-Eluting Stent Era

**DOI:** 10.1155/2019/3270132

**Published:** 2019-07-01

**Authors:** Chengbin Zheng, Jeehoon Kang, Kyung Woo Park, Jung-Kyu Han, Han-Mo Yang, Hyun-Jae Kang, Bon-Kwon Koo, Hyo-Soo Kim

**Affiliations:** Department of Internal Medicine and Cardiovascular Center, Seoul National University Hospital, Seoul, Republic of Korea

## Abstract

**Objectives:**

The aim of our study was to investigate the predictors of target lesion revascularization (TLR) and to compare the in-stent restenosis (ISR) progression rates of different 2nd-generation drug-eluting stents (DES).

**Background:**

The predictors of early and late TLR after 2nd-generation DES implantation have not been fully evaluated.

**Methods:**

We analyzed 944 stented lesions from 394 patients who had at least two serial follow-up angiograms, using quantitative coronary angiography (QCA) analysis. The study endpoints were TLR and the velocity of diameter stenosis (DS) progression.

**Results:**

TLR occurred in 58 lesions (6.1%) during the first angiographic follow-up period and 23 de novo lesions (2.4%) during the following second interval. Independent predictors for early TLR were diabetes mellitus (DM) (HR 2.58, 95% CI 1.29–5.15, p=0.007), previous percutaneous coronary intervention (PCI) (HR 2.41, 95% CI 1.03–5.65, p=0.043), and postprocedure DS% (HR 1.08, 95% CI 1.05–1.11, p<0.001, per 1%), while predictors of late TLR were previous PCI (HR 9.43, 95% CI 2.58-34.52, p=0.001) and serum C-reactive protein (CRP) (HR 1.60, 95% CI 1.28-2.00, p<0.001). The ISR progression velocity (by DS%) was 12.1 ±21.0%/year and 3.7 ±10.1%/year during the first and second follow-up periods, respectively, which had no significant difference (p>0.05) between the four types of DESs.

**Conclusions:**

Our data showed that predictors for TLR may be different at different time intervals. DM, pervious PCI, and postprocedure DS could predict early TLR, while previous PCI and CRP level could predict late TLR. Contemporary DESs had similar rates of ISR progression rates.

**Trial Registration:**

This study was retrospectively registered and approved by the institutional review board of Seoul National University Hospital (no. 1801–138-918).

## 1. Introduction

In-stent restenosis (ISR), which is thought to be mostly caused by neointimal hyperplasia (NIH), was an important medical problem in the era of bare-metal stents (BMS) [1]. Subsequent intravascular ultrasound (IVUS) and quantitative coronary angiography (QCA) studies further strengthened the view that the main mechanism of ISR after BMS implantation was intrastent NIH [2, 3] with a biphasic change of lumen loss in the first 6 months and NIH regression between 6 months and 1–3 years after BMS implantation [4, 5]. Therefore, late TLR after BMS implantation was not a common phenomenon. 

Compared to BMS, DES significantly reduced the rates of ISR and TLR [6, 7]. However, some studies mentioned a “late catch-up” phenomenon after the 1st generation DES implantation [8–14]. The “late catch-up” phenomenon suggests that the mechanism and rate of neointimal formation may be different at different time intervals. Thus, we can assume that the predictors of TLR may also vary from time to time. One clinical study suggested that the risk factors for late TLR were similar to those of early TLR [15]. However, this study only included patients who received sirolimus eluting stent (SES) implantation and was limited by the profound selection bias that favored the use of BMS [16]. At present, there are few studies on predictors of both early and late TLR based on contemporary 2nd generation DESs; moreover, the predictors are not consistent.

In this study, we analyzed stented lesions treated with four types of contemporary 2nd generation DESs, using longitudinal QCA analysis. We observed the progression of stented lesions and identified the independent predictors of early and late TLR. Additionally, we compared the ISR progression rates of the four types of 2nd generation DESs.

## 2. Methods

### 2.1. Study Design and Population

**Figure 1:**
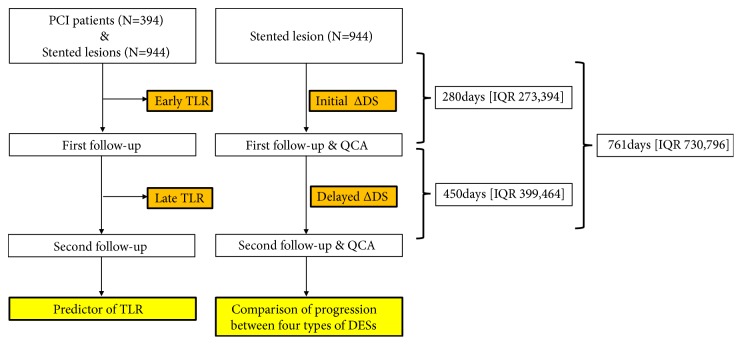
Flowchart of the study.

**Figure 2:**
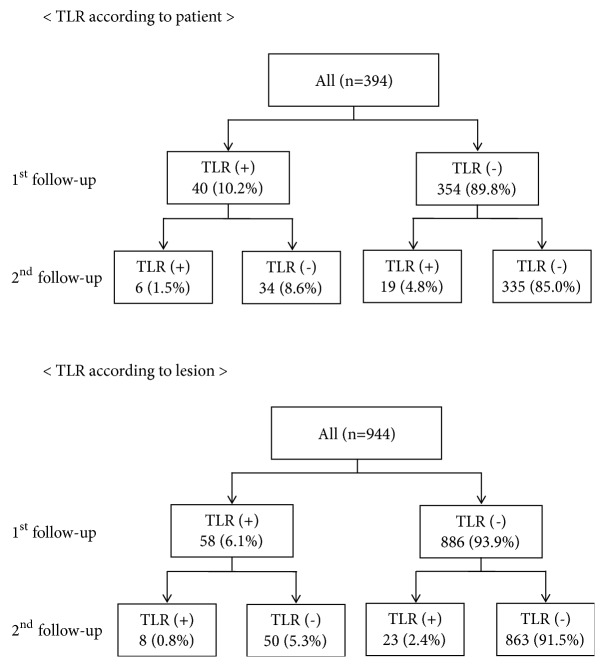
Incidence of target lesion revascularization.

We enrolled patients who underwent previous percutaneous coronary intervention (PCI) in our institute from serial stent registries. These registries included four types of 2nd generation DESs: cobalt chromium everolimus-eluting stent (CoCr-EES), platinum chromium everolimus-eluting stent (PtCr-EES), zotarolimus-eluting stent (ZES), and Biolimus-eluting stent (BES). Follow-up coronary angiography (CAG) was recommended at 9 to 12 months after PCI according to the protocol of the individual registries (this is not mandatory). In the patients who consented to and received the first follow-up angiogram, second follow-up angiogram was recommended at 24 months. During the study period (July 2008 to March 2013), 3,365 patients were enrolled in various stent registries in Seoul National University Hospital. Among these population, 3170 patients (94.2%) were treated with 2^nd^ generation DESs and 1545 patients (45.9%) received at least two serial angiographic follow-ups. 944 lesions from 394 patients (11.7%) were performed in longitudinal QCA analysis. We performed a sensitivity analysis to check the possibility of selection bias, as in the following table, showing that the baseline demographics were similar between the entire parent population and our study population (Supplementary Table 1). Finally, 394 patients and 944 lesions were analyzed. The study flowchart is shown in Figure 1. 58 lesions in 40 patients were needed for TLR at the first angiographic follow-up (early TLR: 6.1%, 10.2%). And 23 lesions in 19 patients which are non-TLR at the first follow-up are needed for late TLR at the second angiographic follow-up (late TLR: 2.4%, 4.8%) (Figure 2). The study was approved by the ethics committee and institutional review board and was conducted according to the principles of the Declaration of Helsinki. All patients provided written, informed consent for participation in the registry.

### 2.2. Procedure and Data Collection

PCI procedure was performed with standard methods. Aspirin 300mg and clopidogrel 300-600mg were administered to all patients before intervention and all patients were given unfractionated heparin to achieve an activated clotting time of 250 seconds or more. The drugs, devices, and intervention techniques (i.e., usage of glycoprotein IIb/IIIa inhibitors, predilatation devices, type of DES, stenting techniques, and imaging guidance) used during the procedure were all determined by experienced operators. Dual antiplatelet therapy was recommended for all patients after DES implantation.

Clinical and procedural data were collected by clinical research coordinators who were unaware of the purpose of the study. Angiographic images at baseline and two serial follow-up periods saved in DICOM format for review and further analyses.

### 2.3. Quantitative Coronary Angiography

Quantitative analysis was performed by specialized QCA technicians who were unaware of the purpose of this study and the type of stent used for treatment at the Seoul National University Hospital Cardiovascular Clinical Research Center Angiographic Core Lab. The parameters of angiography were measured using the CAAS II QCA system (Pie Medical, Maastricht, the Netherlands). After calibration with the guiding catheter, we measured the reference vessel diameter and the minimal luminal diameter (MLD) both in-stent and in-segment at baseline and two serial angiographic follow-ups. All measurements were performed on angiograms recorded after intracoronary administration of nitroglycerine.

### 2.4. Study Endpoints and Definitions

In our study, major adverse cardiac events (MACE) were defined as a composite of all cause death, any myocardial infarction, TLR, and stent thrombosis. TLR was defined as any repeat percutaneous intervention of the target lesion or bypass surgery of the target vessel performed for restenosis or other complication of the target lesion. In this cohort, TLR was performed if angiography during follow-up showed a diameter stenosis ≥50% with at least one of the following: (1) history of recurrent angina pectoris, presumably related to the target vessel; (2) objective signs of ischemia at rest or during exercise test by electrocardiogram, presumably related to target vessel; (3) abnormal test results of invasive functional diagnostic test (fractional flow reserve); or (4) a TLR with a diameter stenosis ≥70%, even in the absence of the aforementioned ischemic signs or symptoms. The study endpoints were independent predictors of TLR (early TLR, defined as TLR during the first follow-up period, and late TLR defined as TLR during the period from the first follow-up to the second follow-up) and the velocity of DS% progression during the follow-up periods. TLR was defined as repeat PCI within the stent or the 5mm borders proximal or distal to the stent. DS% was defined as 100% minus the ratio of MLD and diameter of the reference segment. Early delta DS% indicated the difference in DS% between angiogram immediately after index procedure and the first follow-up angiogram, and delayed delta DS% denoted the changes of DS% between the first and second follow-up. Depending on the time interval, we converted early and delayed delta DS% into rates (early DS%/year & delayed DS%/year). All measurements were performed for both in-stent and in-segment.

### 2.5. Statistical Analysis

Continuous variables are expressed as mean ± standard deviation. Student's t-test or one-way analysis of variance was used for comparison of continuous variables, and we analyzed categorical variables using the chi-square test (or the Fisher exact test when any expected count was <5 for a 2×2 table) test. To determine the independent predictors of early and late TLR, a Cox proportional hazard model was used. Variables included in a Cox multivariate regression analysis were age, sex, body mass index, HTN, DM, dyslipidemia, current smoking, family history of coronary artery disease, previous PCI, chronic renal failure (creatinine clearance level < 60ml/min, stage 3 or higher), multivessel disease, left ventricular dysfunction, hemoglobin, CRP as clinical factors and lesion type B2/C, reference vessel diameter, lesion length, preprocedure DS%, and postprocedure DS% in-stent as lesion-related factors. Statistical analysis was performed with SPSS (version 23.0), and a value of P values less than 0.05 was considered statistically significant.

## 3. Results

From July 2008 to March 2013, a total of 394 patients with 944 lesions were enrolled in the study. The mean follow-up duration from baseline to the first and second angiography was 325±90 days and 772 ±133 days, respectively. The study flowchart is shown in Figure 1.

### 3.1. Baseline Characteristics

The mean age of our study population was 65.5±10.4 years, 73.4% were male patients, 69.8% had hypertension (HTN), and 33.8% had DM. The mean reference vessel diameter of the lesions was 2.92 ±0.52 mm and mean length of lesions was 27.7 ±17.3 mm. Baseline patient and lesion characteristics of the study population are summarized in Supplementary Table 2. Laboratory findings at the follow-up periods are shown in Supplementary Table 3.

### 3.2. Incidence and Predictors of Target Lesion Revascularization

The incidence of all events is shown in Supplementary Table 4. The cumulative incidence of TLR is shown in Figure 2. TLR was performed for 58 lesions (6.1%) in 40 patients (10.2%) at the first angiographic follow-up period and for 23 lesions (2.4%) in 19 patients (4.8%) at the second follow-up period. TLR in this cohort included ischemic driven TLR and TLR during routine angiographic follow-up. Ischemia driven TLR accounted for 15.5% and 30.4% at 1st follow-up and 2nd follow-up, respectively (Supplementary Table 5), and information on TLR methods was described in Supplementary Table 6. In the baseline characteristics, previous PCI and chronic renal failure were more common in patients who experienced early TLR; the hemoglobin level was lower in these patients. Lesion characteristics showed that postprocedural MLD was smaller and postprocedural DS% was larger in the early TLR group (Table 1). However, no discrepant factors were found between the late TLR and nonlate TLR group (Table 2). Regarding independent predictors of TLR, multivariate analysis results showed chronic renal failure (HR 2.37, 95% CI 0.94–5.98, p=0.068) and hemoglobin (HR 0.94, 95% CI 0.77–1.16, p=0.586) could not predict early TLR, although significant differences were shown in the baseline analysis. However, DM (HR 2.58, 95% CI 1.29–5.15, p=0.007), previous PCI (HR: 2.41, 95% CI 1.03–5.65, p=0.043), and postprocedure DS% (HR 1.08, 95% CI 1.05–1.11, p<0.001, per DS of 1%) were independent predictors of early TLR (Table 3). Conversely, independent predictors of late TLR included previous PCI (HR 9.43, 95% CI 2.58-34.52, p=0.001) and CRP (HR 1.60, 95% CI 1.28–2.00, p<0.001) (Table 4).

### 3.3. Angiographic Outcome

In our study, 944 lesions were treated using four types of 2nd generation DES: CoCr-EES, ZES, BES, and PtCr-EES; each accounted for 25.8%, 32.8%, 17.6%, and 23.7%, respectively. There were no significant differences in both reference vessel diameter (p=0.054 using ANOVA) and lesion length (p=0.247 using ANOVA) between the four types of DES. QCA analysis of 944 lesions showed that the mean angiographic DS% before the procedure was 74.8% ±15.7%; it decreased to 11.8% ±8.5% (in-stent DS%) and 21.5% ± 11.6% (in-segment DS%) after the procedure (Supplementary Table 2). The mean MLD also showed no significant difference between the four DES groups at all the different points in time (initial state, postprocedure, at the first angiographic follow-up, and at the second angiographic follow-up) (Table 5). The MLD cumulative probability curve of the four groups is shown in Supplementary Figure 1. Both early delta DS% and delayed delta DS% were also similar between the four DESs (Early delta DS%: 9.5 ±16.0%, 10.3 ±15.2%, 9.5 ±14.4%, 11.7 ±17.6%, p=0.204 using ANOVA, delayed delta DS%: 4.8 ±14.1%, 3.7 ±9.9%, 5.3 ±11.5%, 5.1 ±11.1%, p=0.486 using ANOVA, for CoCr-EES, ZES, BES, and PtCr-EES, respectively.)

Regarding ISR progression, the early delta DS%/year was 12.12% ±20.97%/year, and delayed delta DS%/year was 3.68% ±10.10%/year, showing that the delayed ISR progression rate was about 30% of the early progression rate. Between the four types of DES, both the early ISR progression rate and delayed ISR progression rate showed no significant difference (early ISR progression rate: 12.2% ±20.9%/year, 12.2% ±18.6%/year, 14.3% ±29.8%/year, 10.4% ±15.7%/year, p=0.525; delayed ISR progression rate: 4.1% ±12.8%/year, 2.4% ±8.1% /year, 4.0% ±8.3%/year, 4.8% ±10.5%/year, p=0.205) (Table 5).

## 4. Discussion

In this study, the cumulative incidence rate for early TLR was 6.1%, and 2.4% for late TLR in stented lesions, during the overall median follow-up period of 772 days. The rate of ISR progression of early and late was 12.1 ±21.0 %/year and 3.7 ±10.1%/year, respectively. Additionally, independent predictors of early TLR were DM, previous PCI, and postprocedure DS%, while those for late TLR were previous PCI and high serum CRP level, differing from each other. Additional analysis showed that the rate of early DS%/year and delayed DS%/year were all similar (p>0.05) among the four types of DES.

### 4.1. Mechanism of Restenosis after DES Implantation

Restenosis is a progressive phenomenon and the specific mechanism at different time intervals of ISR is difficult to explain exactly. Since entering the era of support, the two mechanisms recoil and vascular remodeling had been almost cancelled with the advent of stents compared to simple balloon angioplasty and NIH became the primary mechanism for restenosis [17]. ISR is primarily a nonspecific inflammatory response to vessel wall injury and the injured tissue reacts via an inflammatory process that leads to NIH, eventually leading to lumen narrowing. Regardless of the exact pathophysiology, ISR is the end result of endothelial injury caused by stent deployment and foreign materials left at the deployment site [16, 18–20]. Goto K et al. retrospectively analyzed 298 ISR lesions using IVUS data. The main findings of this study were that NIH was an important mechanism of ISR even in the 2nd generation DES era [21]. Compared with BMS, although the drug and polymer of DES counteract the excessive NIH, which greatly reduced the incidence of ISR, there was a late catch-up phenomenon [22]. Due to drugs and polymers, DES prolongs the healing time of endothelial compared to BMS. This may be the reason why the incidence of early and late events is different. This is also the main reason for prolonging DAPT duration after entering the DES era. However, the exact mechanisms of ISR in DES remain unclear. The possible mechanisms of restenosis after DES that can be considered at present are biological factors (drug resistance, hypersensitivity), mechanical factors (stent underexpansion, nonuniform stent strut distribution, stent fracture, nonuniform drug elution/deposition, and polymer peeling), and technical factors (barotrauma outside stented segment, stent gap, and residual uncovered atherosclerotic plaques) [22]) DESs are constantly improving, but ISR is still an important difficulty to overcome.

### 4.2. Predictors of In-Stent Restenosis

The “late catch-up” phenomenon mentioned by previous studies suggests that the mechanism and rate of neointimal formation may be different at different time intervals after the 1st generation DES implantation [8–14]. After implantation of DES, the stent will gradually be covered by neointima, which may last for about one year due to the effect of the drug and polymer. Assuming that one year is the benchmark, the predictors may be different within one year and one year later. In our study, the incidence of early TLR in our study was 6.1%, while the rate of late TLR was 2.4%. Our data also showed that independent predictors for late TLR differed from the predictors of early TLR. Previous studies had suggested that the predictors of early TLR are MLD poststent implantation, saphenous vein graft, DM, RCA disease, family history of CAD, multivessel disease, stent diameter, etc., while insulin-treated DM, younger age, elevated serum hs-CRP levels, the first generation DES, stent fracture, stent diameter, and stent length are predictive predictors of late TLR [16, 23–25]. Some studies focused on early TLR which was evaluated at 8-12 months after PCI [25, 26], while other studies focused on late TLR, which was evaluated at 12-24 months after index PCI [23, 24]. Due to the distinct characteristics of different cohorts, this should be analyzed in an identical cohort to compare the different predictors of TLR at different points in time. Also, most of the stents in these studies are first generation DES, which are known to have a high rate of TLR compared to that with contemporary DESs [26, 27]. With the constant improvement of DES, the predictors of ISR may be different in new generation DES. Compared to the above studies, the population of our study was all treated with 2nd generation DES, and both the predictors of early and late TLR were analyzed in a single cohort. From the result of our study, the independent predictors of early TLR were previous PCI, DM, and postprocedure DS, while previous PCI and CRP were independent predictors for late TLR. The predictors in our study can be explained as follows. A history of PCI, which was a consistent predictor of early and late TLR, is known to directly represent the overall atherosclerotic risk of an individual and is a general risk factor for vascular atherosclerosis and restenosis [28]. As for DM, some putative mechanisms, including more aggressive intimal hyperplasia, higher inflammatory response, higher coagulability, and endothelial dysfunction, have been considered to be probable causes of a high rate of early TLR in patients with DM after stent implantation [29]. Procedural factors also determine restenosis, such as stent underexpansion and incomplete apposition. In our study, postprocedural DS was only a predictor of early TLR, which could explain why procedural factors are important to prevent restenosis in the early phase. CRP is recognized as an important marker of vascular wall inflammation and as a strong predictor of adverse cardiovascular events. It may play an important role in the pathogenesis of NIH after coronary stenting [30]. Choi et al.'s [16] study also suggested that high levels of CRP is an independent risk factor for late TLR after DES implantation. Our findings are on the same line as this study. High CRP levels mean chronic inflammatory state and promote NIH, which can persist to late stages and affect the ISR progression.

Collectively, we analyzed the predictors of early and late TLR in patients treated with 2nd generation DES in a single cohort, which could discriminate the distinct predictors of TLR in different phases. Quantitative analysis for progression of ISR was also performed making our findings more convincing.

### 4.3. ISR Progression in 2nd Generation DES

For further analysis, we compared the ISR progression rate using QCA analysis. The early delta DS%/year was 3.3-fold larger than the delayed delta DS%/year, implying that ISR progression is more rapid in the early phase. Interestingly, a previous study by Kang et al. analyzed the natural progression of atherosclerotic plaques [31]. This study showed that the natural progression rate of nonstented lesion was 2.19%/year, which was smaller than stented lesions. From these results, we can conclude that stented lesions are more susceptible to restenosis, compared to the progression of natural atherosclerotic lesions.

### 4.4. Limitation

Frist, due to the retrospective nature of our study, there could have been a selection bias in patient selection. We compared the baseline clinical characteristics with the total parent population whom received PCI during the study period, and we found minimal difference between the two populations. However, we cannot complete deny possibilities of other selection bias within our study population. Second, we did not perform imaging analysis using IVUS, which may enable assessment of the cause of restenosis. Lastly, the data used in analysis in this study were based on a relatively small sample and were collected from a single center.

## 5. Conclusion

Our data suggested that the factors of DM, previous PCI, and postprocedure in-stent DS% were predictors of early TLR, and the predictors for late TLR were previous PCI and high level serum CRP. Predictors for TLR may be different at different time intervals in 2nd generation DES era. There was no difference in the rate of DS progression in stented lesion between four types of contemporary DESs.

## Figures and Tables

**Table 1:** Baseline patients and lesion characteristics according to early TLR.

Clinical Factors	Early TLR(-)	Early TLR(+)	P
	(354 patients)	(40 patients)	
Age (years old)	65.3±10.4	66.9±11.0	0.369
BMI (kg/m^2^)	24.8±2.9	23.9±3.3	0.073
Male sex, n (%)	265 (74.9)	24 (60.0)	0.068
Previous PCI, n (%)	38 (10.7)	10 (25.0)	0.018
Diabetes mellitus, n (%)	114 (32.2)	19 (47.5)	0.078
Hypertension, n (%)	246 (69.5)	29 (72.5)	0.833
CRF, n (%)	137 (38.7)	25 (62.5)	0.006
Dyslipidemia, n (%)	225 (63.6)	23 (57.5)	0.562
Current smoking, n (%)	95 (26.8)	9 (22.5)	0.689
FHx of CAD, n (%)	44 (12.4)	6 (15.0)	0.807
LV ejection fraction (%)	60.3±8.3	57.3 ± 10.6	0.119
Clinical diagnosis			0.127
(i) Silent ischemia, n (%)	21 (5.9)	0 (0)	
(ii) Stable angina, n (%)	209 (59.0)	23 (57.5)	
(iii) Unstable angina, n (%)	71 (20.1)	7 (17.5)	
(iv) STEMI, n (%)	22 (6.2)	2 (5.0)	
(v) NSTEMI, n (%)	31 (8.8)	8 (20.0)	
Multi Vessel disease, n (%)	255 (72.0)	31 (77.5)	0.576

*Laboratory tests*			

WBC (10^9^/L)	6.9±2.4	6.5±1.7	0.332
Hemoglobin (g/dl)	13.6±1.8	12.9±2.0	0.018
Creatinine(mg/dl)	1.05±0.44	1.39±1.60	0.183
GFR(ml/min/1.73m^2^)	70.8±24.0	57.5±28.2	0.015
Total cholesterol (mg/dl)	157±38	159±45	0.845
Triglyceride (mg/dl)	140±86	120±66	0.193
LDL (mg/dl)	97±35	94±44	0.666
HDL (mg/dl)	42±11	44±12	0.302
CRP (mg/dl)	0.40±1.17	0.37±0.84	0.870

*Medication at discharge*			

(i) Aspirin, n (%)	353 (99.7)	40 (100.0)	1.000
(ii) Clopidogrel, n (%)	353 (99.7)	40 (100.0)	1.000
(iii) DAPT, n (%)^#^	352 (99.4)	40 (100.0)	1.000
(iv) Beta blocker, n (%)	251 (70.9)	28 (70.0)	1.000
(v) Statin, n (%)	337 (95.2)	39 (97.5)	1.000
(vi) CCB, n (%)	75 (21.2)	11 (27.5)	0.418
(vii) ACEI, n (%)	85 (24.0)	16 (40.0)	0.035
(viii) ARB, n (%)	134 (37.9)	19 (47.5)	0.237

*Medication at first follow-up*			

(i) Aspirin, n (%)	351 (99.2)	40 (100.0)	1.000
(ii) Clopidogrel, n (%)	346 (97.7)	39 (97.5)	1.000
(iii) DAPT, n (%)^#^	343 (96.9)	39 (97.5)	1.000
(iv) Beta blocker, n (%)	266 (75.1)	28 (70.0)	0.451
(v) Statin, n (%)	352 (99.4)	40 (100.0)	1.000
(vi) CCB, n (%)	79 (22.3)	8 (20.0)	0.842
(vii) ACEI, n (%)	36 (10.2)	8 (20.0)	0.106
(viii) ARB, n (%)	145 (41.0)	13 (32.5)	0.395

*Medication at second follow-up*			

(i) Aspirin, n (%)	324 (91.5)	38 (95.0)	0.758
(ii) Clopidogrel, n (%)	300 (84.7)	37 (92.5)	0.239
(iii) DAPT, n (%)^#^	272 (76.8)	35 (87.5)	0.159
(iv) Beta blocker, n (%)	256 (72.3)	25 (62.5)	0.200
(v) Statin, n (%)	349 (98.6)	39 (97.5)	0.476
(vi) CCB, n (%)	68 (19.2)	9 (22.5)	0.674
(vii) ACEI, n (%)	28 (7.9)	7 (17.5)	0.070
(viii) ARB, n (%)	137 (38.7)	13 (32.5)	0.495

Lesion factors	Early TLR(-)	Early TLR(+)	
	(886 lesions)	(58 lesions)	

Treated coronary location			0.854
(i) LAD, n (%)	411 (46.4)	28 (48.3)	
(ii) LCX, n (%)	218 (24.6)	16 (27.6)	
(iii) RCA, n (%)	255 (28.8)	14 (24.1)	
Bifurcation lesion, n (%)	308 (34.8)	25 (43.1)	0.204
Calcified lesion			0.004
(i) None, n (%)	451 (51.2)	26 (44.8)	
(ii) Mild, n (%)	155 (17.6)	4 (6.9)	
(iii) Moderate, n (%)	114 (12.9)	7 (12.1)	
(iv) Severe, n (%)	161 (18.3)	21 (36.2)	
Tortuous lesion			0.465
(i) None, n (%)	819 (92.9)	56 (96.6)	
(ii) Mild, n (%)	24 (2.7)	0 (0)	
(iii) Moderate, n (%)	26 (2.9)	2 (3.4)	
(iv) Severe, n (%)	13 (1.5)	0 (0)	
Angulation lesion, n (%)			0.305
(i) None (<45°), n (%)	821 (93.3)	56 (98.2)	
(ii) Moderate (45°<90°), n (%)	37 (4.2)	1 (1.8)	
(iii) Extreme n (>90°), n (%)	22 (2.5)	0 (0)	
Thrombus in lesion, n (%)	29 (3.3)	0 (0)	0.291
Ostial lesion, n (%)	169 (19.1)	14 (24.1)	0.390
Ulceration, n (%)	2 (0.2)	3 (5.2)	0.002
Aneurysm, n (%)	10 (1.1)	0 (0)	1.000
Lesion type			0.142
(i) A, n (%)	92 (10.4)	8 (13.8)	
(ii) B1, n (%)	214 (24.2)	8 (13.8)	
(iii) B2, n (%)	52 (5.9)	7 (12.1)	
(iv) C, n (%)	499 (56.3)	34 (58.6)	
Type B2/C lesion, n (%)	551 (64.3)	41 (71.9)	0.305
Reference vessel diameter (mm)	2.92±0.51	2.78±0.66	0.100
Lesion length (mm)	27.7±17.1	28.8±19.4	0.647
Pre-procedure MLD (mm)	0.74±0.50	0.68±0.49	0.385
Pre-procedure DS (%)	74.7±15.7	75.9±15.5	0.569
Post-procedure MLD, in- stent (mm)	2.49±0.43	2.26±0.48	<0.001
Post-procedure MLD, in-segment (mm)	2.13±0.51	1.85±0.57	<0.001
Post-procedure DS, in- stent (mm)	11.4±8.1	18.0±11.2	<0.001
Post-procedure DS, in-segment (mm)	21.0±11.3	29.9±13.6	<0.001

TLR, target lesion revascularization; BMI, body mass index; kg, kilogram; PCI, percutaneous coronary intervention; CRF, chronic renal failure; FHx, family history; CAD, coronary artery disease; LV, left ventricle; STEMI, ST-elevation myocardial infarction; NSTEMI, non-ST-elevation myocardial infarction; WBC, white blood cell; GFR, glomerular filtration rate; LDL, low density lipoprotein cholesterol; HDL, high density lipoprotein cholesterol; CRP, C-reactive protein; DAPT, dual antiplatelet therapy; CCB, calcium channel blockers; ACEI, angiotensin converting enzyme inhibitors; ARB, angiotensin II receptor blockers; LAD, left anterior descending; LCX, left circumflex; RCA, right coronary artery; MLD, minimal lumen diameter; DS, diameter stenosis.

^#^ DAPT: combination of aspirin and clopidogrel.

**Table 2:** Baseline patients and lesion characteristics according to late TLR.

Clinical Factors	Late TLR(-)	Late TLR(+)	P
	(335 patients)	(19 patients)	
Age (years old)	65.5±10.3	62.8±11.2	0.272
BMI (kg/m^2^)	24.9±2.9	24.3±2.8	0.429
Male sex, n (%)	249 (74.3)	16 (84.2)	0.488
Previous PCI, n (%)	33 (9.9)	5 (26.3)	0.061
Diabetes mellitus, n (%)	108 (32.2)	6 (31.6)	1.000
Hypertension, n (%)	232 (69.3)	14 (73.7)	0.879
CRF, n (%)	129 (38.5)	8 (42.1)	0.811
Dyslipidemia, n (%)	213 (63.6)	12 (63.2)	1.000
Current smoking, n (%)	87 (26.0)	8 (42.1)	0.201
FHx of CAD, n (%)	44 (13.1)	0 (0)	0.223
LV ejection fraction (%)	60.2±8.4	62.5±6.9	0.300
Clinical diagnosis			0.632
(i) Silent ischemia, n (%)	19 (5.7)	2 (10.5)	
(ii) Stable angina, n (%)	197 (58.8)	12 (63.2)	
(iii) Unstable angina, n (%)	67 (20.0)	4 (21.1)	
(iv) STEMI, n (%)	21 (6.3)	1 (5.3)	
(v) NSTEMI, n (%)	31 (9.3)	0 (0.0)	
Multi Vessel disease, n (%)	245 (73.1)	10 (52.6)	0.066

*Laboratory tests*			

WBC (10^9^/L)	6.9±2.4	7.4±2.2	0.354
Hemoglobin (g/dl)	13.6±1.8	13.7±2.0	0.840
Creatinine(mg/dl)	1.04±0.43	1.19±0.63	0.303
GFR(ml/min/1.73m^2^)	70.9±24.1	69.6±24.4	0.864
Total cholesterol (mg/dl)	157±38	157±44	0.982
Triglyceride (mg/dl)	141±88	118±38	0.283
LDL (mg/dl)	97±35	102±38	0.576
HDL (mg/dl)	42±11	39±8	0.277
CRP (mg/dl)	0.34±0.92	1.44±3.27	0.200

*Medication at discharge*			

(i) Aspirin, n (%)	334 (99.7)	19 (100.0)	1.000
(ii) Clopidogrel, n (%)	334 (99.7)	19 (100.0)	1.000
(iii) DAPT, n (%)^#^	333 (99.4)	19 (100.0)	1.000
(vi) Beta blocker, n (%)	237 (70.7)	14 (73.7)	1.000
(v) Statin, n (%)	318 (94.9)	19 (100.0)	0.612
(vi) CCB, n (%)	66 (19.7)	9 (47.4)	0.008
(vii) ACEI, n (%)	82 (24.5)	3 (15.8)	0.581
(viii) ARB, n (%)	128 (38.2)	6 (31.6)	0.634

*Medication at first follow-up*			

(i) Aspirin, n (%)	332 (99.1)	19 (100.0)	1.000
(ii) Clopidogrel, n (%)	328 (97.9)	18 (94.7)	0.360
(iii) DAPT, n (%)^#^	325 (97.0)	18 (94.7)	0.460
(vi) Beta blocker, n (%)	252 (75.2)	14 (73.7)	1.000
(v) Statin, n (%)	333 (99.4)	19 (100.0)	1.000
(vi) CCB, n (%)	72 (21.5)	7 (36.8)	0.153
(vii) ACEI, n (%)	35 (10.4)	1 (5.3)	0.706
(viii) ARB, n (%)	136 (40.6)	9 (47.4)	0.634

*Medication at second follow-up*			

(i) Aspirin, n (%)	307 (91.6)	17 (89.5)	0.669
(ii) Clopidogrel, n (%)	282 (84.2)	18 (94.7)	0.329
(iii) DAPT, n (%)^#^	256 (76.4)	16 (84.2)	0.581
(vi) Beta blocker, n (%)	244 (72.8)	12 (63.2)	0.429
(v) Statin, n (%)	330 (98.5)	19 (100.0)	1.000
(vi) CCB, n (%)	62 (18.5)	6 (31.6)	0.225
(vii) ACEI, n (%)	28 (8.4)	0 (0)	0.382
(viii) ARB, n (%)	127 (37.9)	10 (52.6)	0.230

Lesion factors	Late TLR(-)	Late TLR(+)	
	(863 lesions)	(23 lesions)	

Treated coronary location			0.482
(i) LAD, n (%)	397 (46.0)	14 (60.9)	
(ii) LCX, n (%)	215 (24.9)	3 (13.0)	
(iii) RCA, n (%)	249 (28.9)	6 (26.1)	
Bifurcation lesion, n (%)	298 (34.5)	10 (43.5)	0.382
Calcified lesion			0.109
(i) None, n (%)	437 (50.9)	14 (60.9)	
(ii) Mild, n (%)	154 (17.9)	1 (4.3)	
(iii) Moderate, n (%)	113 (13.2)	1 (4.3)	
(vi) Severe, n (%)	154 (17.9)	7 (30.4)	
Tortuous lesion			0.761
(i) None, n (%)	797 (92.8)	22 (95.7)	
(ii) Mild, n (%)	24 (2.8)	0 (0)	
(iii) Moderate, n (%)	25 (2.9)	1 (4.3)	
(vi) Severe, n (%)	13 (1.5)	0 (0)	
Angulation lesion, n (%)			0.428
(i) None (<45°), n (%)	798 (93.1)	23 (100.0)	
(ii) Moderate (45°<90°), n (%)	37 (4.3)	0 (0)	
(iii) Extreme n (>90°), n (%)	22 (2.6)	0 (0)	
Thrombus in lesion, n (%)	26 (3.0)	3 (13.0)	0.291
Ostial lesion, n (%)	165 (19.1)	4 (17.4)	1.000
Ulceration, n (%)	2 (0.2)	0 (0)	1.000
Aneurysm, n (%)	10 (1.2)	0 (0)	1.000
Lesion type			0.132
(i) A, n (%)	87 (10.1)	5 (21.7)	
(ii) B1, n (%)	206 (23.9)	8 (34.8)	
(iii) B2, n (%)	50 (5.8)	2 (8.7)	
(vi) C, n (%)	491 (56.9)	8 (34.8)	
Type B2/C lesion, n (%)	541 (64.9)	10 (43.5)	0.059
Reference vessel diameter (mm)	2.93±0.51	2.89±0.53	0.713
Lesion length (mm)	27.7±17.1	25.3±20.2	0.561
Pre-procedure MLD (mm)	0.74±0.50	0.75±0.59	0.926
Pre-procedure DS (%)	74.7±15.7	74.9±17.3	0.938
Post-procedure MLD, in- stent (mm)	2.49±0.43	2.55±0.40	0.509
Post-procedure MLD, in-segment (mm)	2.12±0.51	2.26±0.55	0.216
Post-procedure DS, in- stent (mm)	11.4±8.1	11.3±9.6	0.955
Post-procedure DS, in-segment (mm)	21.0±11.3	20.5±10.9	0.825

TLR, target lesion revascularization; BMI, body mass index; kg, kilogram; PCI, percutaneous coronary intervention; CRF, chronic renal failure; FHx, family history; CAD, coronary artery disease; LV, left ventricle; STEMI, ST-elevation myocardial infarction; NSTEMI, non-ST-elevation myocardial infarction; WBC, white blood cell; GFR, glomerular filtration rate; LDL, low density lipoprotein cholesterol; HDL, high density lipoprotein cholesterol; CRP, C-reactive protein; DAPT, dual antiplatelet therapy; CCB, calcium channel blockers; ACEI, angiotensin converting enzyme inhibitors; ARB, angiotensin II receptor blockers; LAD, left anterior descending; LCX, left circumflex; RCA, right coronary artery; MLD, minimal lumen diameter; DS, diameter stenosis.

^#^ DAPT: combination of aspirin and clopidogrel.

**Table 3:** Predictors of early TLR.

Clinical Factors	Adjusted Hazard Ratio	95% CI	P
Age (years old)	0.96	0.92-1.00	0.070
BMI (≥23kg/m^2^)	0.78	0.37-1.61	0.494
Gender (male)	0.58	0.26-1.30	0.187
Previous PCI	2.41	1.03-5.65	0.043
Diabetes mellitus	2.58	1.29-5.15	0.007
Hypertension	0.70	0.31-1.60	0.397
CRF	2.37	0.94-5.98	0.068
Dyslipidemia	0.56	0.27-1.14	0.108
Current smoking	0.92	0.37-2.30	0.858
FHx of CAD	0.98	0.35-2.71	0.967
LV dysfunction (<40%)	2.01	0.41-9.76	0.387
Multivessel disease	1.20	0.52-2.81	0.669
Hemoglobin (g/dl)	0.94	0.77-1.16	0.586
CRP (mg/dl)	1.03	0.73-1.44	0.889
Type B2/C lesion	1.46	0.64-3.37	0.371
Reference vessel diameter (mm)	0.77	0.44-1.35	0.366
Lesion length (mm)	0.99	0.97-1.01	0.208
Pre-procedure DS (%)	1.01	0.98-1.04	0.546
Post-procedure DS%, in- stent (mm)	1.08	1.05-1.11	<0.001

TLR, target lesion revascularization; CI, confidence interval; BMI, body mass index; PCI, percutaneous coronary intervention; CRF, chronic renal failure; FHx, family history; CAD, coronary artery disease; LV, left ventricle; CRP, C-reactive protein; DS, diameter stenosis.

**Table 4:** Predictors of late TLR.

Clinical Factors	Adjusted Hazard Ratio	95% CI	P
Age (years old)	0.97	0.90-1.04	0.359
BMI (≥23kg/m^2^)	1.68	0.40-6.94	0.477
Gender (male)	2.39	0.38-14.95	0.351
Previous PCI	9.43	2.58-34.52	0.001
Diabetes mellitus	1.56	0.47-5.21	0.472
Hypertension	0.87	0.25-3.11	0.835
CRF	1.45	0.30-7.04	0.641
Dyslipidemia	0.70	0.20-2.48	0.579
Current smoking	2.56	0.78-8.40	0.122
FHx of CAD	NA	NA	0.974
LV dysfunction (<40%)	NA	NA	0.988
Multivessel disease	0.42	0.12-1.41	0.159
Hemoglobin (g/dl)	1.19	0.77-1.85	0.429
CRP (mg/dl)	1.60	1.28-2.00	<0.001
Type B2/C lesion	0.22	0.04-1.11	0.067
Reference vessel diameter (mm)	0.82	0.31-2.21	0.696
Lesion length (mm)	1.03	0.99-1.08	0.165
Pre-procedure DS (%)	0.97	0.94-1.08	0.142
Post-procedure DS%, in- stent (mm)	1.01	0.95-1.08	0.703

TLR, target lesion revascularization; CI, confidence interval; BMI, body mass index; PCI, percutaneous coronary intervention; CRF, chronic renal failure; FHx, family history; CAD, coronary artery disease; LV, left ventricle; CRP, C-reactive protein; DS, diameter stenosis.

**Table 5:** Lesion characteristics and DS% progression rate according to types of DESs.

Clinical Factors	Overall	CoCr-EES	ZES	BES	PtCr-EES	P
	(N=944)	(N=244)	(N=310)	(N=166)	(N=224)	
Reference vessel diameter (mm)	2.92±0.52	2.97±0.56	2.91±0.52	2.96±0.49	2.85±0.48	0.054
Lesion length (mm)	27.73±17.25	26.04±16.45	28.97±17.98	26.56±15.96	28.93±18.05	0.247
Pre-procedure MLD (mm)	0.73±0.50	0.76±0.53	0.65±0.51	0.87±0.44	0.71±0.46	0.755
MLD in-segment (mm)						
(i) Post-procedure	2.11±0.52	2.13±0.55	2.08±0.52	2.13±0.50	2.11±0.51	0.906
(ii) 1^st^ follow-up	1.97±0.58	1.97±0.59	1.99±0.57	1.95±0.58	1.96±0.58	0.726
(iii) 2^nd^ follow-up	1.96±0.52	2.01±0.55	1.94±0.52	1.96±0.49	1.93±0.52	0.180
MLD in-stent (mm)						
(i) Post-procedure	2.48±0.44	2.49±0.47	2.47±0.43	2.47±0.42	2.48±0.44	0.852
(ii) 1^st^ follow-up	2.19±0.60	2.19±0.62	2.20±0.59	2.21±0.59	2.15±0.60	0.782
(iii) 2^nd^ follow-up	2.13±0.55	2.16±0.61	2.12±0.54	2.17±0.50	2.09±0.54	0.480
Pre-procedure DS (%)	74.8±15.7	74.4±16.0	77.0±16.6	70.6±13.7	75.1±14.9	0.431
DS% in-segment						
(i) Post-procedure	21.5±11.6	22.9±12.5	21.1±11.8	21.4±10.8	20.8±11.0	0.075
(ii) 1^st^ follow-up	26.2±16.5	27.3±17.0	25.0±15.9	27.5±17.6	25.8±15.9	0.649
(iii) 2^nd^ follow-up	27.3±15.0	27.9±15.8	26.2±15.3	27.7±12.6	28.1±15.2	0.624
DS% in-stent						
(i) Post-procedure	11.8±8.5	13.0±8.4	10.5±8.8	12.6±9.0	11.7±7.4	0.460
(ii) 1^st^ follow-up	22.0±17.5	22.4±17.9	20.8±16.4	21.8±18.3	23.3±17.9	0.454
(iii) 2^nd^ follow-up	23.7±15.5	24.0±17.8	22.8±15.3	22.9±12.3	25.3±15.5	0.346
Acute gain (mm)						
(i) In-segment	1.38±0.59	1.37±0.58	1.42±0.63	1.26±0.48	1.40±0.60	0.731
(ii) In-stent	1.74±0.54	1.73±0.53	1.81±0.58	1.61±0.47	1.75±0.51	0.395
Initial ΔDS	4.85±15.13	4.71±16.53	4.00±14.80	6.33±14.32	5.10±14.59	0.457
In-segment (%)						
Initial ΔDS	10.28±15.89	9.52±16.02	10.27±15.15	9.46±14.43	11.72±17.63	0.204
In-stent (%)						
Delayed ΔDS	3.66±11.87	3.65±13.52	2.82±11.53	4.04±9.95	4.55±11.81	0.264
In-segment (%)						
Delayed ΔDS	4.59±11.60	4.81±14.08	3.73±9.87	5.27±11.49	5.10±11.11	0.486
In-stent (%)						
Initial DS%/year in-segment	6.08±20.77	6.48±22.87	4.75±17.49	10.22 ±29.61	4.45±13.00	0.841
Initial DS%/year in-stent	12.12±20.97	12.23±20.92	12.15±18.60	14.34±29.76	10.36±15.74	0.525
Delayed DS%/year in-segment	2.98±9.84	2.65±9.93	2.09±9.57	3.26±7.89	4.30±11.16	0.130
Delayed DS%/year in-stent	3.68±10.10	4.11±12.83	2.39±8.13	4.02±8.30	4.77±10.46	0.205

CoCr-EES, cobalt chromium everolimus-eluting stent; ZES, zotarolimus-eluting stent; BES, Biolimus-eluting stent: PtCr-EES, platinum chromium everolimus-eluting stent; MLD, minimal lumen diameter; DS, diameter stenosis.

## Data Availability

The data used to support the findings of this study are available from the corresponding author upon request.

## Supplementary Materials

Supplementary Materials Figure 1. Cumulative probability curve of MLD (postprocedure, first angiographic follow-up, and second angiographic follow-up). Table 1. Baseline demographic and clinical characteristics of the current study population and total patent population. Table 2. Baseline patients and lesions characteristics. Table 3. Laboratory findings at the follow-up periods. Table 4. Clinical events during the follow-up period. Table 5. Ischemic driven and angiographic driven TLR. Table 6. TLR method.
