# Supplementary material for: The Predictors of Target Lesion Revascularization and Rate of In-Stent Restenosis in the Second-Generation Drug-Eluting Stent Era
Source: J Interv Cardiol. 2019 Jul 1;2019:3270132. doi: 10.1155/2019/3270132 (PMC6739790; doi:10.1155/2019/3270132)

Supplementary Appendix

The predictors of target lesion revascularization in second-generation drug-eluting stent era

Chengbin Zheng, Jeehoon Kang et al.

1. **Supplementary Tables**
2. **Supplementary Figure**

Supplementary Table 1. Baseline demographic and clinical characteristics of the current study population and total patent population.

	Current study population (n=394)	Total parent population (n=3365)	P value
**Age (years old)**	65.5±10.4	65.5±10.4	1.000
**BMI (kg/m^2)**	24.7±2.9	24.5±3.1	0.223
**Male sex, n (%)**	289 (73.4)	2383 (70.8)	0.668
**Previous PCI, n (%)**	48 (12.2)	612 (18.2)	0.011
**Diabetes mellitus, n (%)**	133 (33.8)	1322 (39.3)	0.150
**Hypertension, n (%)**	275 (69.8)	2217 (65.9)	0.488
**Dyslipidemia, n (%)**	248 (62.9)	2294 (68.2)	0.350
**CRF, n (%)**	162 (41.1)	1323 (39.3)	0.651
**Current Smoking, n (%)**	104 (26.4)	778 (23.1)	0.258
**FHx of CAD, n (%)**	52 (13.2)	398 (11.8)	0.484
**LV ejection fraction, (%)**	60.0±8.6	57.4±10.4	<0.001
**Clinical diagnosis**			0.002
**- Silent ischemia, n (%)**	21 (5.3)	169 (5.0)	
**- Stable angina, n (%)**	232 (58.9)	1630 (48.4)	
**- Unstable angina, n (%)**	78 (19.8)	809 (24.0)	
**- STEMI, n (%)**	24 (6.1)	362 (10.8)	

**- NSTEMI, n (%)**	39 (9.9)	391 (11.6)	
**Vessel disease**			0.363
**- 1 Vessel disease, n (%)**	108 (27.4)	1051 (31.2)	
**- 2 Vessel disease, n (%)**	149 (37.8)	1143 (34.0)	
**- 3 Vessel disease, n (%)**	137 (34.8)	1171 (34.8)	

BMI, body mass index; PCI, percutaneous coronary intervention; CRF, chronic renal failure; FHx, family history; CAD, coronary artery disease; LV, left ventricle; STEMI, ST-elevation myocardial infarction; NSTEMI, non ST-elevation myocardial infarction.

Supplementary Table 2. Baseline patients and lesions characteristics

**Patient character (N=394)**	**Value**
**Age (years old)**	65.5±10.4
**BMI (kg/m^2)**	24.7±2.9
**Male sex, n (%)**	289 (73.4)
**Previous PCI, n (%)**	48 (12.2)
**Previous CABG, n (%)**	7 (1.8)
**Previous MI, n (%)**	29 (7.4)
**Previous CHF, n (%)**	3 (0.8)
**Diabetes mellitus, n (%)**	133 (33.8)
**- No control, n (%)**	16 (4.1)
**- Dietary, n (%)**	6 (1.5)
**- Oral, n (%)**	102 (25.9)
**- Insulin, n (%)**	20 (5.1)

**Hypertension, n (%)**	275 (69.8)
**CRF, n (%)**	162 (41.1)
**Dyslipidemia, n (%)**	248 (62.9)
**Smoking, (%)***	44.7 / 26.4 / 27.9
**FHx of CAD, n (%)**	52 (13.2)
**LV Ejection fraction, (%)**	60.0±8.6
**Clinical diagnosis, (%)†**	5.3 / 58.9 / 19.8 / 6.1 / 9.9
**Number of Vessel disease, (%)‡**	27.4 / 37.8 / 34.8
Laboratory tests	
**WBC (10^9/L)**	6.9±2.3
**Hemoglobin (g/dl)**	13.5±1.8
**Creatinine(mg/dl)**	1.08±0.66
**GFR (ml/min)**	69.3±24.8
**CrCl (ml/min)**	67.6±23.1
**HbA1c (%)**	6.79±1.21
**Total cholesterol (mg/dl)**	158±39
**Triglyceride (mg/dl)**	138±84
**LDL (mg/dl)**	97±36
**HDL (mg/dl)**	42±11
**CRP(mg/dl)**	0.40±1.13
Medication at discharge	
**- Aspirin, n (%)**	393 (99.7)
**- Clopidogrel, n (%)**	393 (99.7)
**- DAPT, n (%)#**	392 (99.5)
**- Beta blocker, n (%)**	279 (70.8)
**- Statin, n (%)**	376 (95.4)
**- CCB, n (%)**	86 (21.8)
**- ACEI, n (%)**	101 (25.6)

**- ARB, n (%)**	153 (38.8)
Medication at first follow-up	
**- Aspirin, n (%)**	391 (99.2)
**- Clopidogrel, n (%)**	385 (97.7)
**- DAPT, n (%)#**	382 (97.0)
**- Beta blocker, n (%)**	294 (74.6)
**- Statin, n (%)**	392 (99.5)
**- CCB, n (%)**	87 (22.1)
**- ACEI, n (%)**	44 (11.2)
**- ARB, n (%)**	158 (40.1)
Medication at second follow-up	
**- Aspirin, n (%)**	362 (91.9)
**- Clopidogrel, n (%)**	337 (85.5)
**- DAPT, n (%)#**	307 (77.9)
**- Beta blocker, n (%)**	281 (71.3)
**- Statin, n (%)**	388 (98.5)
**- CCB, n (%)**	77 (19.5)
**- ACEI, n (%)**	35 (8.9)
**- ARB, n (%)**	150 (38.1)
**Lesion character (N=944)**	
**Stent/person (n)**	2.4±1.6
**Second generation DES (%)**	100
**Stent type**	
**- CoCr-EES (%)**	25.8
**- ZES (%)**	32.8
**- BES (%)**	17.6
**- PtCr-EES (%)**	23.7
**Lesion type**	100
**- A (%)**	10.6
**- B1 (%)**	23.5

**- B2 (%)**	6.3
**- C (%)**	56.5
**Type B2/C lesion (%)**	62.7
**Treated coronary location**	
**- LM (%)**	8.8
**- LAD (%)**	37.7
**- Proximal LAD (%)**	22.7
**- Mid LAD (%)**	8.7
**- Distal LAD (%)**	1.9
**- Diagonal 1 (%)**	3.7
**- Diagonal 2 (%)**	0.7
**- LCX (%)**	24.8
**- Proximal LCX (%)**	11.2
**- Distal LCX (%)**	8.6
**- Ramus (%)**	2.0
**- OM1 (%)**	2.6
**- OM2 (%)**	0.3
**- RCA (%)**	28.5
**- Proximal RCA (%)**	17.4
**- Mid RCA (%)**	5.0
**- Distal RCA (%)**	4.7
**- PDA (%)**	0.7
**- PL branch (%)**	0.7
**- SVG (%)**	0.2
**Bifurcation lesion (%)**	35.3
**Calcified lesion (moderate & severe) (%)**	32.1
**Tortuous lesion (%)**	6.9
**Angulation lesion (%)**	6.4
**Thrombus in lesion (%)**	3.1
**Ostial lesion (%)**	19.4

**Ulceration (%)**	0.5
**Aneurysm (%)**	1.1
**Previously treated lesion (%)**	1.7
**Stent diameter (mm)**	3.03±0.44
**Stent length (mm)**	25.4±6.0
**Pre-procedure MLD (mm)**	0.73±0.50
**Pre-procedure DS (%)**	74.8±15.7
**Reference Vessel Diameter (mm)**	2.92±0.52
**Lesion length (mm)**	27.7±17.3
**Post-procedure MLD (in-stent) (mm)**	2.48±0.44
**Post-procedure MLD (in-segment) (mm)**	2.11±0.52
**Post-procedure DS (in-stent) (%)**	11.8±8.5
**Post-procedure DS (in-segment) (%)**	21.5±11.6
Restenosis pattern at first angiographic follow-up	
**- 0-No Restenosis (%)**	89.1
**- 1-Focal articulation or Gap (%)**	0.3
**- 2-Focal in stent (stent body) (%)**	3.1
**- 3-Focal in stent (stent proximal edge) (%)**	2.5
**- 4-Focal in stent (stent distal edge) (%)**	0.4
**- 5-Multiple focal in segment (<5mm from edge) (%)**	0.8
**- 6-Diffuse in stent (%)**	1.3
**- 7-Diffuse proliferative (%)**	0.1
**- 8-Complete stent occlusion (%)**	0.8
Restenosis pattern at second angiographic follow-up	
**- 0-No Restenosis (%)**	83.2
**- 1-Focal articulation or Gap (%)**	0.0
**- 2-Focal in stent (stent body) (%)**	1.6
**- 3-Focal in stent (stent proximal edge) (%)**	2.0
**- 4-Focal in stent (stent distal edge) (%)**	1.3
**- 5-Multiple focal in segment (<5mm from edge) (%)**	0.3

**- 6-Diffuse in stent (%)**	1.1
**- 7-Diffuse proliferative (%)**	0.2
**- 8-Complete stent occlusion (%)**	0.4

BMI, body mass index; kg, kilogram; PCI, percutaneous coronary intervention; CABG, coronary artery bypass graft surgery; MI, Myocardial infarction; CHF, Congestive heart failure; CRF, chronic renal failure; FHx, family history; CAD, coronary artery disease; LV, left ventricle; WBC, white blood cell; GFR, glomerular filtration rate; CrCl, creatinine clearance; HbA1c, hemoglobin A1c; LDL, low density lipoprotein cholesterol; HDL, high density lipoprotein cholesterol; CRP, C-reactive protein; DAPT, dual antiplatelet therapy; CCB, calcium channel blockers; ACEI, angiotensin converting enzyme inhibitors; ARB, angiotensin II receptor blockers; DES, drug eluting stent; CoCr-EES, cobalt chromium everolimus-eluting stent; ZES, zotarolimus-eluting stent; BES, Biolimus- eluting stent: PtCr-EES, platinum chromium everolimus-eluting stent; LM, left main; LAD, left anterior descending; LCX, left circumflex; OM, obtuse marginal; RCA, right coronary artery; PDA, posterior descending artery; PL, posterolateral; SVG, saphenous vein graft; MLD, minimal lumen diameter; DS, diameter stenosis.

* Smoking: Never smoker / Current smoker / Ex-smoker

† Clinical diagnosis: Silent ischemia / Stable angina / Unstable angina / non ST-segment elevation myocardial infarction / ST-segment elevation myocardial infarction

‡ Vessel disease: 1 Vessel disease / 2 Vessel disease / 3 Vessel disease

# DAPT: Combination of aspirin and Clopidogrel.

Supplementary Table 3. Laboratory findings at the follow-up periods

	Baseline	1st follow-up	2nd follow-up
**WBC (10^9/L)**	6.9±2.3	6.0±1.5	5.9±1.5
**Hemoglobin (g/dl)**	13.5±1.8	13.2±1.8	13.4±1.6
**Creatinine(mg/dl)**	1.08±0.66	1.02±0.70	1.07±0.97
**Total cholesterol (mg/dl)**	158±39	129±27	129±25
**Triglyceride (mg/dl)**	138±84	115±51	113±54
**LDL (mg/dl)**	97±36	66±22	65±20
**HDL (mg/dl)**	42±11	44±12	47±12
**CRP(mg/L)**	0.40±1.13	0.14±0.43	0.23±0.85

WBC, white blood cell; LDL, low density lipoprotein cholesterol; HDL, high density lipoprotein cholesterol; CRP, C-reactive protein

Supplementary Table 4. Clinical events during the follow-up period

	First follow-up (N=394)	Second follow-up (N=394)
**MACE, n (%)**	40 (10.2)	59 (15.0)
**- All death, n (%)**	0 (0)	1 (0.3)
**- All MI, n (%)**	2 (0.5)	3 (0.8)
**- TLR, n (%)**	40 (10.2)	59 (15.0)
**- Stent thrombosis, n (%)**	0 (0)	0 (0)

MACE, major adverse cardiac events; MI, myocardial infarction; TLR, target lesion revascularization.

Supplementary Table 5. Ischemic driven and angiographic driven TLR

	**Early TLR (+) (N=58)**	**Late TLR (+) (N=23)**
**Ischemic driven TLR, n (%)**	9 (15.5)	7 (30.4)
**Angiographic driven TLR, n (%)**	49 (84.5)	16 (69.6)

TLR, target lesion revascularization.

Supplementary Table 6. TLR method

	**Early TLR (+) (N=58)**	**Late TLR (+) (N=23)**
**POBA, n (%)**	30 (51.7)	10 (43.5)
**DEB, n (%)**	20 (34.5)	9 (39.1)
**DES, n (%)**	8 (13.8)	4 (17.4)
**- Same type of DES, n (%)**	2 (3.4)	2 (8.7)
**- Different type of DES, n (%)**	6 (10.3)	2 (8.7)

TLR, target lesion revascularization; POBA, plain Old Balloon Angioplasty; DEB, drug eluting balloon; DES, drug eluting stent.

Supplementary Figure 1. Cumulative probability curve of MLD (post procedure, first angiographic follow-up, second angiographic follow-up)

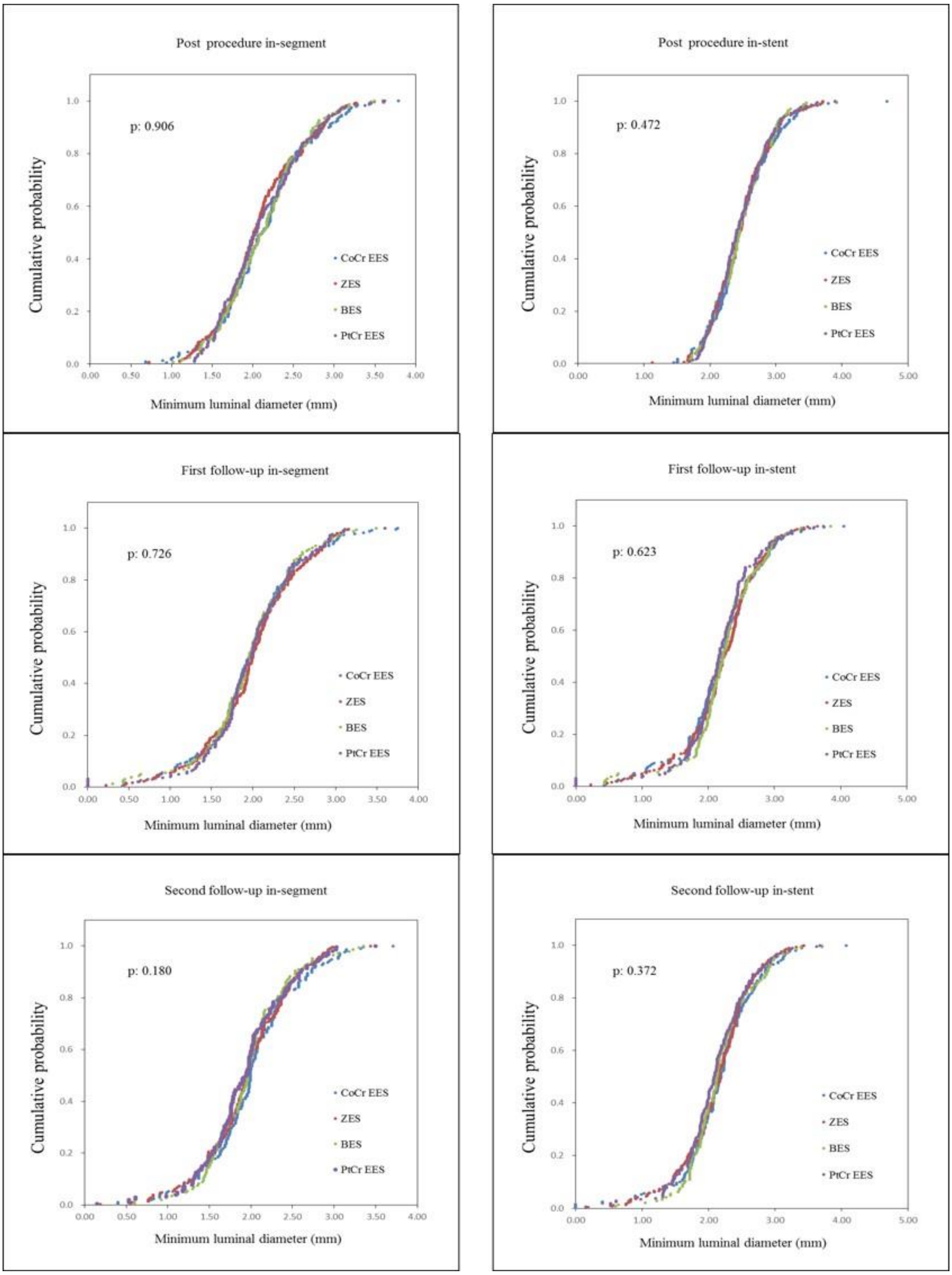

Supplement: Supplementary Materials — Figure 1. Cumulative probability curve of MLD (postprocedure, first angiographic follow-up, and second angiographic follow-up). Table 1. Baseline demographic and clinical characteristics of the current study population and total patent population. Table 2. Baseline patients and lesions characteristics. Table 3. Laboratory findings at the follow-up periods. Table 4. Clinical events during the follow-up period. Table 5. Ischemic driven and angiographic driven TLR. Table 6. TLR method. [file 3270132.f1.pdf]
